# A versatile cohesion manipulation system probes female reproductive age-related egg aneuploidy

**DOI:** 10.1038/s43587-025-00997-w

**Published:** 2025-11-03

**Authors:** Jiyeon Leem, Tom Lemonnier, Ani Khutsaidze, Lei Tian, Xiaojun Xing, Suxia Bai, Timothy Nottoli, Binyam Mogessie

**Affiliations:** 1 Department of Molecular, Cellular and Developmental Biology, Yale University, New Haven, CT USA; 2 Yale Genome Editing Center, Yale University School of Medicine, New Haven, CT USA; 3 Department of Comparative Medicine, Yale Genome Editing Center, Yale University School of Medicine, New Haven, CT USA; 4 Department of Obstetrics, Gynecology and Reproductive Sciences, Yale University School of Medicine, New Haven, CT USA

**Keywords:** Meiosis, Centromeres, Cohesion, Chromosome segregation, Ageing

## Abstract

Female reproductive aging is accompanied by a sharp increase in egg aneuploidy rates. Premature loss of chromosome cohesion proteins and early separation of chromosomes are thought to cause high aneuploidy rates during maternal aging. However, because cohesion loss occurs gradually throughout a woman’s reproductive lifespan, and because cytoskeletal defects alone can lead to chromosomal abnormalities, the main causes of the rapid rise in aneuploidy at older reproductive ages are still unclear. In this study, we created a versatile and tunable cohesion manipulation system that enables rapid, dose-dependent degradation of the meiotic cohesin REC8 in live mouse oocytes. By coupling this system with quantitative high-resolution live imaging, we directly observed cohesion protein behavior during meiosis and tested the longstanding threshold model of aneuploidy development. Our results show that premature sister chromatid separation sharply increases only when REC8 levels drop below a critical threshold, supporting the idea of a nonlinear, vulnerability-triggering cohesion limit. We also used our system to examine how other age-related issues, such as cytoskeletal disruption and partial centromere dysfunction, can exacerbate chromatid separation in the context of weakened cohesion. This work provides a tractable oocyte platform for modeling and dissecting the multifactorial mechanisms driving female reproductive age-related egg aneuploidy.

## Abstract (web summary)

To study pathways that lead to aneuploidy during aging, the authors provide a system that enables cohesion protein depletion in mouse oocytes, mimicking effects that occur during aging. They uncover a threshold for cohesion loss driving chromosome errors and show that actin and centromere defects amplify aneuploidy.

## Main

Chromosome segregation is critical for eukaryotic cell division. When fertilizable eggs are formed from progenitor oocytes, a specialized form of meiotic cell division separates the chromosomes. Accurate chromosome segregation in mammalian oocytes and eggs is driven by a meiotic spindle machinery that is assembled from microtubules^1^ and is reinforced with spindle actin filaments that mediate chromosome−microtubule attachments^2,3^. Unlike somatic cell mitosis or sperm meiosis, female meiotic chromosome segregation is remarkably susceptible to errors^4,5^. Egg aneuploidy, a cellular state of incorrect chromosome numbers, that arises from oocyte chromosome segregation errors typically leads to chromosomal abnormalities associated with miscarriages, infertility and genetic conditions, including intellectual disabilities (for example, Down syndrome)^4–6^. Importantly, the incidence of egg aneuploidy increases almost exponentially at advanced female reproductive ages^4–6^, which dramatically impacts the quality of eggs and leads to poor reproductive outcomes. With a record-high maternal age at first pregnancy^7–10^, declining fertility and a rapidly aging global human population^11–16^, understanding the molecular origins of female reproductive age-related egg aneuploidy is a critical step toward addressing one of the most pressing clinical and socioeconomic challenges posed by delayed childbearing and population aging^9^.

Female reproductive age-related loss of chromosome cohesion is thought to underlie high chromosome segregation error rates in oocytes^4–6^. Consistently, in mice^17–19^ and humans^20^, REC8, SGO2 and other cohesin components gradually become depleted with advancing maternal age. However, given the progressive nature of cohesion weakening as well as actin and microtubule cytoskeletal defects that are common in aged oocytes^2,21^, it is conceivable that cohesion depletion-independent causes of chromosome segregation errors trigger the sharp rise in oocyte aneuploidy during aging. The lack of tools for direct and temporally resolved manipulation of chromosome cohesion in oocytes has remained a major hurdle in uncovering these root causes of age-related oocyte aneuploidy.

## Results

We reasoned that discovering novel causes of age-related egg aneuploidy necessitates tools for controlled generation of aging-like premature chromatid separation events in young oocytes. To achieve this, we innovated a versatile experimental system that enables direct observation of REC8, a meiosis-specific cohesin subunit^22,23^, in live oocytes and allows its destruction via proteolysis targeting chimera (PROTAC)^24–26^ or single variable domain antibody (nanobody)^27–29^ technologies.

**Extended Data Fig. 1:**
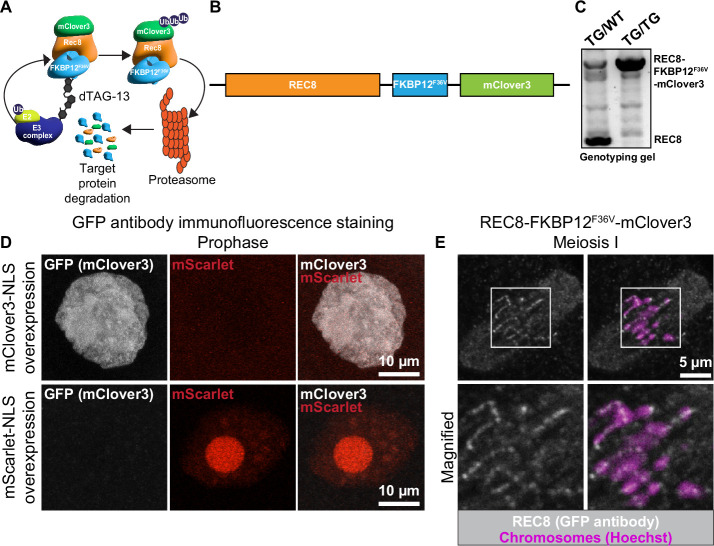
Generation of REC8-FKBP12^F36V^-mClover3 mice as a versatile cohesion manipulation system. (**A**) Graphical description of the principle of dTAG-13 proteolysis targeting chimera-mediated endogenous protein degradation. (**B**) Graphical description of gene arrangements in REC8-FKBP12^F36V^-mClover3 knockin mice. Boxes depicting genes are drawn to scale. (**C**) A representative genotyping agarose gel analysis of REC8-FKBP12^F36V^-mClover3 mouse genomic DNA using oligonucleotide pairs designed to distinguish between wild-type and knockin REC8 alleles. (**D**) Representative maximum intensity projected immunofluorescence images showing that GFP antibodies specifically recognize mClover3 but not mScarlet in mClover3-NLS or mScarlet-NLS expressing prophase-arrested wild-type oocytes. (**E**) Representative maximum intensity projected immunofluorescence images of REC8 (detected with GFP antibody) and chromosomes in metaphase I-stage oocytes isolated from REC8-FKBP12^F36V^-mClover3 mice. Boxes represent regions of interest magnified in lower panels. Source data

We previously showed that the dTAG system, a protein degradation tool wherein the PROTAC dTAG-13 targets FKBP12^F36V^-chimeric proteins for proteasomal degradation^30,31^ (Extended Data Fig. 1a), can successfully be used to rapidly degrade exogenously expressed actin mutants in mammalian oocytes^32^. In addition, we demonstrated that the TRIM-Away method of rapid protein degradation can be coupled with green fluorescent protein (GFP) nanobodies for efficient removal of fluorescence-tagged endogenous proteins^33^. To extend these approaches to targeted degradation of chromosome cohesion proteins and generation of prematurely separated chromatids, we applied CRISPR knockin technology and engineered mice in which REC8 is C-terminally tagged with FKBP12^F36V^−mClover3 (Extended Data Fig. 1b,c).

**Fig. 1:**
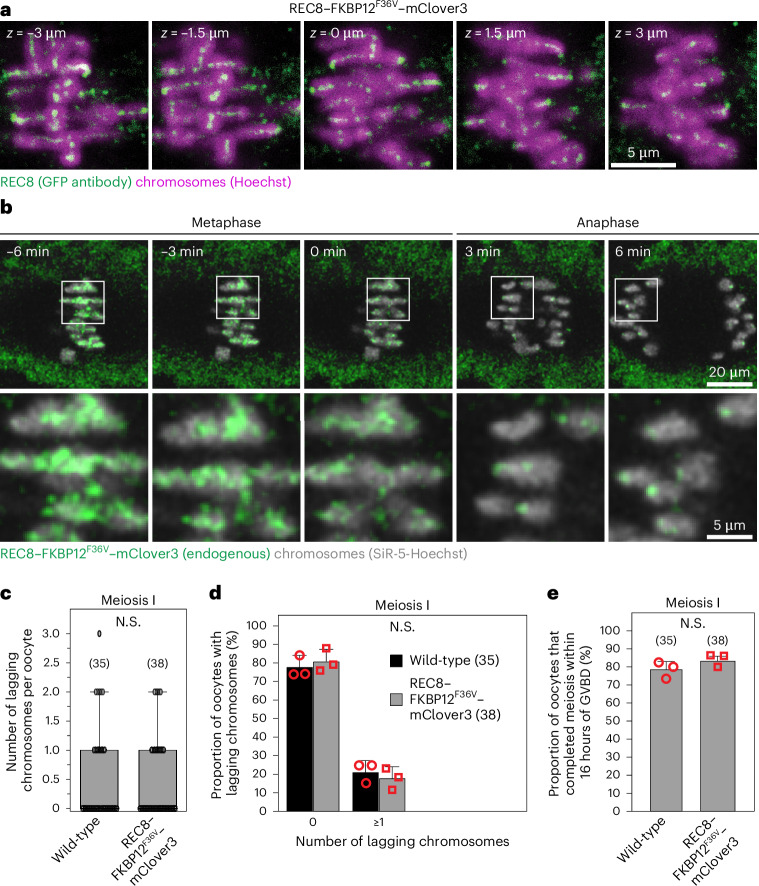
REC8−FKBP12^F36V^−mClover3 knockin mice enable high-resolution live imaging of endogenous REC8 dynamics during oocyte meiosis. **a**, Representative confocal immunofluorescence microscopy images of REC8 (marked with GFP antibodies) and chromosomes (Hoechst) in metaphase I-stage oocytes of a REC8−FKBP12^F36V^−mClover3 mouse. Single confocal sections spaced 1.5 µm apart are shown. **b**, Images from a representative high-resolution time-lapse movie of REC8−FKBP12^F36V^−mClover3 (endogenous REC8) and chromosomes (SiR-5-Hoechst) showing chromosome arm-specific removal of REC8 cohesin (and its retention at centromeric regions) during anaphase of meiosis I. **c**, Quantification of the number of anaphase I lagging chromosomes per cell in wild-type and REC8−FKBP12^F36V^−mClover3 oocytes. Data points represent individual oocytes. **d**, Quantification of the proportion of oocytes with zero or more anaphase I lagging chromosomes in wild-type and REC8−FKBP12^F36V^−mClover3 oocytes. **e**, Quantification of the proportion of oocytes that completed meiosis within 16 hours of GVBD in wild-type and REC8−FKBP12^F36V^−mClover3. Data are from three independent experiments. Statistical significance was evaluated using Student’s *t*-test (**c**–**e**). The number of analyzed oocytes is indicated in brackets and italics. GVBD, germinal vesicle breakdown; N.S., non-significant values. Source data

We validated that this endogenous protein-tagging approach did not interfere with REC8 protein localization and function by immunostaining homozygous REC8−FKBP12^F36V^−mClover3 mouse oocytes with anti-GFP antibodies that cross-react with mClover3 (Extended Data Fig. 1d). Three-dimensional high-resolution immunofluorescence microscopy assays demonstrated that anti-GFP antibodies recognize REC8 protein along the arms and centromeric regions of homologous chromosomes (Fig. 1a, Extended Data Fig. 1e and Supplementary Video 1), confirming that fusion to FKBP12^F36V^−mClover3 does not impact its localization. Sequential removal of REC8 cohesin from chromosome arms in meiosis I and from centromeric regions of DNA in meiosis II underpins successive separation of homologous chromosomes and sister chromatids^5,34,35^. High-spatiotemporal-resolution microscopy of SiR-5-Hoechst-labeled chromosomes and REC8−FKBP12^F36V^−mClover3 allowed us, to our knowledge for the first time, to directly visualize this selective REC8 degradation phenomenon in live oocytes undergoing meiosis I chromosome segregation (Fig. 1b and Supplementary Video 2). Consistent with our immunofluorescence microscopy data (Fig. 1a), these observations confirmed that oocyte REC8 cohesin dynamics is unaffected in these engineered mice.

**Extended Data Fig. 2:**
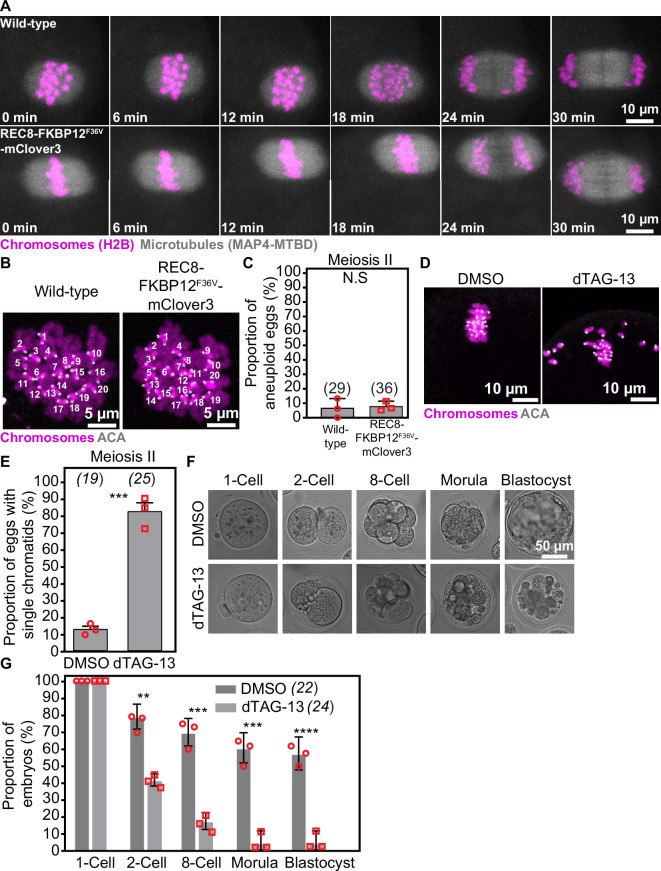
CRISPR-Cas based C-terminal tagging of endogenous REC8 does not disrupt accurate oocyte chromosome alignment and segregation. (**A**) Images from representative time lapse movies of chromosomes (marked with H2B-mScarlet) and meiotic spindles (marked with MAP4 microtubule-binding domain (mNeonGreen-MAP4-MTBD)) in wild-type and REC8-FKBP12^F36V^-mClover3 oocytes. (**B**) Representative metaphase II chromosome spreads with centromeres labeled by ACA staining in wild-type and REC8-FKBP12^F36V^-mClover3 oocytes. (**C**) The percentage of aneuploid eggs matured *in vitro* from wild-type or REC8-FKBP12^F36V^-mClover3 oocytes. (**D**) Representative images of metaphase II-arrested eggs matured *in vitro* from REC8-FKBP12^F36V^-mClover3 oocytes treated with DMSO or dTAG-13 for 1 h and stained with ACA and Hoechst. (**E**) Quantification of the percentage of eggs with prematurely separated sister chromatids following 1-h dTAG-13 treatment during metaphase I followed by washout and progression into meiosis II. (**F**) Representative images of embryos at various stages of preimplantation development following fertilization of DMSO- or dTAG-13-treated REC8-FKBP12^F36V^-mClover3 eggs. (**G**) Quantification of developmental progression from the zygote to blastocyst stage in embryos generated by fertilization of DMSO- or dTAG-13-treated REC8-FKBP12^F36V^-mClover3 eggs. Data are from three independent experiments. Statistical significance was assessed using Student’s t-test. The number of analyzed cells or embryos is indicated in brackets and italics. Source data

We further determined that endogenously tagged REC8 protein maintains its physiological function in chromosome cohesion using quantitative high-resolution live imaging of meiotic spindles and chromosomes^36^ in oocytes isolated from homozygous knockin and wild-type mice. Analyses of chromosome organization and dynamics in these imaging assays showed that C-terminal tagging of endogenous REC8 with FKBP12^F36V^−mClover3 did not interfere with accurate metaphase I and II chromosome alignment, anaphase I chromosome segregation, egg euploidy or timing and efficiency of meiosis completion (Fig. 1c–e, Extended Data Fig. 2a–c and Supplementary Videos 3 and 4). Cohesion dysfunction is associated with premature dissociation of bivalents into univalents (meiosis I) and of sister chromatids into single chromatids (meiosis II)^17,18,20^, which is expected to increase the frequency of chromosome misalignment and missegregation. Our data thus demonstrate that REC8 cohesin function is not compromised in REC8−FKBP12^F36V^−mClover3 homozygous females.

**Fig. 2:**
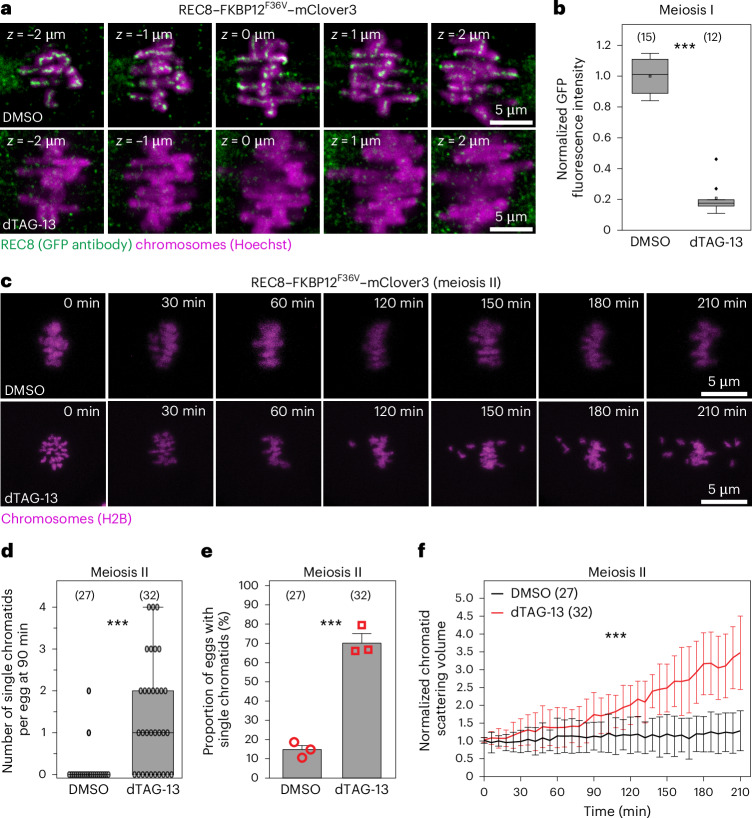
dTAG-13-mediated reduction of endogenous REC8 in REC8−FKBP12^F36V^−mClover3 oocytes rapidly recapitulates premature sister chromatid separation events associated with female reproductive aging. **a**, Endogenous REC8 (labeled with GFP antibody) and metaphase I chromosomes (Hoechst) in DMSO-treated or dTAG-13-treated oocytes isolated from REC8−FKBP12^F36V^−mClover3 mice. Single confocal sections spaced 1 µm apart are shown. **b**, Quantification of REC8 (labeled with GFP antibody) fluorescence intensity in DMSO-treated or dTAG-13-treated metaphase I oocytes isolated from REC8−FKBP12^F36V^−mClover3 mice. **c**, Images from representative time-lapse movies of sister chromatids (H2B−mScarlet) in DMSO-treated or dTAG-13-treated eggs matured in vitro from REC8−FKBP12^F36V^−mClover3 oocytes. *t* = 0 minutes denotes the start of live imaging experiment. **d**, Quantification of the number of single chromatids per egg at 90 minutes of live imaging in DMSO-treated or dTAG-13-treated eggs matured in vitro from REC8−FKBP12^F36V^−mClover3 oocytes. **e**, Quantification of the proportion of cells containing single chromatids in DMSO-treated or dTAG-13-treated eggs matured in vitro from REC8−FKBP12^F36V^−mClover3 oocytes. **f**, Measurements of chromatid scattering volumes from time-lapse movies of DMSO-treated or dTAG-13-treated metaphase II-arrested eggs matured in vitro from REC8−FKBP12^F36V^−mClover3 oocytes. Lines represent mean values, and error bars indicate s.e.m. Data are from three independent experiments. Statistical significance was evaluated using Student’s *t*-test (**b**,**d**,**e**) or two-way ANOVA (**f**). The number of analyzed oocytes is indicated in brackets and italics. Source data

**Extended Data Fig. 3:**
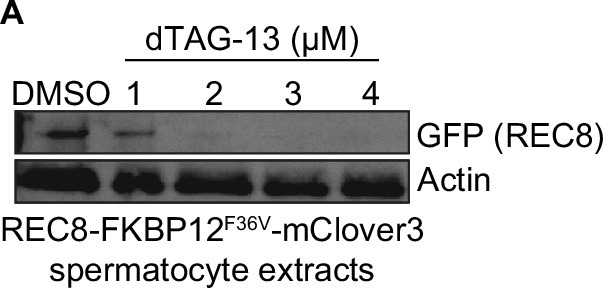
Validation of dTAG-13-mediated REC8 degradation in REC8-FKBP12F36V-mClover3 spermatocytes. (**A**) Western blotting analyses of REC8-FKBP12^F36V^-mClover3 spermatocyte extracts treated with DMSO (control) or varying concentrations of dTAG-13. GFP antibody was used for detection of REC8 protein. Actin was used as loading control. Source data

To test whether endogenous REC8 cohesin in engineered animals can be targeted for rapid degradation via PROTACs, we acutely treated metaphase II-arrested eggs from reproductively young REC8−FKBP12^F36V^−mClover3 females with dimethyl sulfoxide (DMSO) (control) or dTAG-13 (refs. ^30,31^). Using quantitative immunofluorescence microscopy of GFP antibody-stained cells to measure REC8 protein levels, we observed that REC8 cohesin fluorescence intensity was significantly reduced in dTAG-13-treated eggs (Fig. 2a,b). Consistently, western blotting analyses showed robust degradation of REC8 protein in dTAG-13-treated cytosolic extracts of spermatocytes isolated from REC8−FKBP12^F36V^−mClover3 homozygous males (Extended Data Fig. 3a).

We next examined whether PROTAC-mediated targeted degradation of REC8 cohesin can be used to induce aging-like meiotic chromatid separation events in reproductively young females. Here, we leveraged our established quantitative high-resolution live imaging assays of chromosome dynamics^2,3,36^ to track in three dimensions the scattering of prematurely separated sister chromatids. We found that dTAG-13-mediated REC8 cohesin reduction could generate up to four prematurely separated single chromatids within 1.5 hours of treatment (Fig. 2c–f and Supplementary Videos 5 and 6), a process that normally takes years to decades in mice and humans^2,5,20,37–39^. Notably, this rate is consistent with our previously reported measurements of premature chromatid separation in naturally aged eggs^2^. Our quantitative analyses of chromatid separation additionally showed rapid scattering of sister chromatids after 1.5 hours of dTAG-13 treatment (Fig. 2f), which is consistent with sustained degradation of REC8 in the presence of dTAG-13.

To test whether our system can be applied in a biologically relevant setting where premature sister chromatid separation occurs during the completion of meiosis I, we acutely treated metaphase I-stage oocytes with dTAG-13, washed out the compound after 1 hour and allowed the oocytes to progress through anaphase I. We then assessed premature separation of sister chromatids in metaphase II eggs. These experiments confirmed that dTAG-13-mediated REC8 degradation during meiosis I is sufficient to trigger chromatid separation events that manifest in metaphase II (Extended Data Fig. 2d,e), demonstrating the utility of our system for modeling age-related segregation defects in a temporally and developmentally appropriate manner. These results established that experimental reduction of REC8 in engineered REC8−FKBP12^F36V^−mClover3 mice rapidly generates aneuploidy-inducing chromosomal defects that are common in eggs of reproductively older females.

We observed that dTAG-13 treatment for 90 minutes typically induced the separation of 3−4 sister chromatids per oocyte. Although this may appear incomplete compared to systems using TEV protease cleavage^40^, this level of separation is consistent with chromatid separation frequencies observed in naturally aged oocytes^2^. Notably, the dTAG and TRIM-Away systems rely on proteasomal degradation rather than synchronous enzymatic cleavage and, thus, achieve gradual, rather than immediate, depletion of REC8. In extended live imaging experiments, we observed a continued increase in chromatid separation over time, supporting the idea that longer treatments drive more extensive cohesin loss (Fig. 5 and Supplementary Videos 14–16). These findings suggest that chromatid separation scales with the degree and duration of REC8 depletion, highlighting the tunability of our system for modeling progressive cohesion loss.

To assess how acute cohesion weakening affects embryo development, we treated metaphase II-arrested REC8−FKBP12^F36V^−mClover3 oocytes with dTAG-13 for 60 minutes and then thoroughly washed out the compound before fertilizing the oocytes with capacitated sperm. We cultured the resulting embryos for 3 days and monitored their progression through pre-implantation stages. Although both control and dTAG-13-treated oocytes fertilized efficiently and reached the two-cell stage at similar rates, we observed a marked decrease in the number of embryos that advanced to the eight-cell, morula and blastocyst stages in the dTAG-13 group compared to controls (Extended Data Fig. 2f,g). These results show that REC8 degradation in young oocytes impairs developmental potential, likely due to meiotic errors that compromise chromosomal integrity. Although we did not perform graded REC8 depletion in this study, our findings demonstrate that a 60-minute degradation window is sufficient to elicit a measurable functional decline. This experiment establishes a direct link between cohesion loss and reduced developmental capacity and highlights the power of our system to connect molecular defects with fertility-related outcomes. In future work, we will calibrate REC8 degradation levels to model specific stages of maternal aging more precisely.

**Fig. 3:**
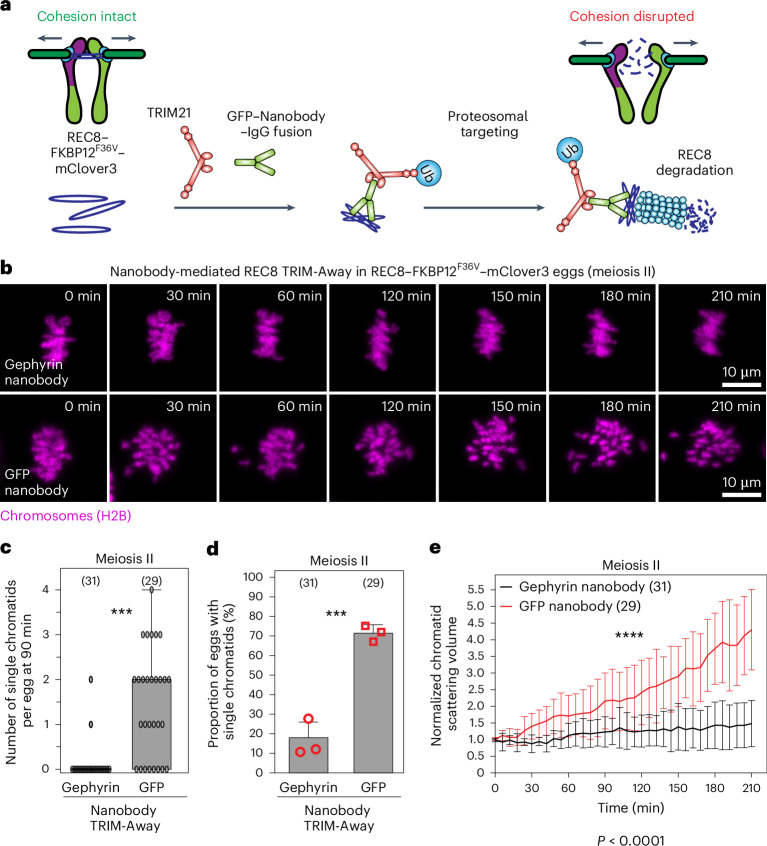
Nanobody-mediated TRIM-Away of endogenous REC8 in REC8−FKBP12^F36V^−mClover3 oocytes accelerates premature separation of sister chromatids. **a**, Graphical description of nanobody-based approach for rapid degradation of endogenous REC8 in REC8−FKBP12^F36V^−mClover3 oocytes. Recognition of mClover3-tagged REC8 by IgG−Fc1 fusion GFP nanobodies in TRIM21-expressing REC8−FKBP12^F36V^−mClover3 eggs induces endogenous REC8 degradation via TRIM-Away and generates prematurely separated sister chromatids. **b**, Images from representative time-lapse movies of sister chromatids (H2B−mScarlet) in Gephyrin or GFP TRIM-Away eggs from REC8−FKBP12^F36V^−mClover3 mice. *t* = 0 minutes denotes the start of live imaging experiment. **c**, Quantification of the number of single chromatids per egg at 90 minutes of live imaging in Gephyrin or GFP TRIM-Away eggs from REC8−FKBP12^F36V^−mClover3 mice. **d**, Quantification of the proportion of cells containing single chromatids in Gephyrin or GFP TRIM-Away eggs from REC8−FKBP12^F36V^−mClover3 mice. **e**, Measurements of chromatid scattering volumes from time-lapse movies of Gephyrin or GFP TRIM-Away metaphase II-arrested eggs from REC8−FKBP12^F36V^−mClover3 mice. Lines represent mean values, and error bars indicate s.e.m. Data are from three independent experiments. Statistical significance was evaluated using Student’s *t*-test (**c**,**d**) or two-way ANOVA (**e**). The number of analyzed oocytes is indicated in brackets and italics. Source data

**Extended Data Fig. 4:**
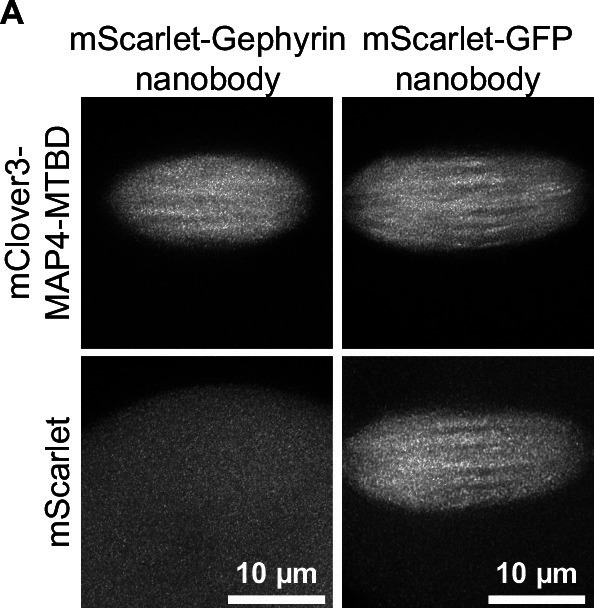
GFP-nanobodies specifically recognize mClover3 protein. (**A**) High-resolution images of live, metaphase II-arrested wild-type mouse eggs co-expressing mClover3-MAP4-MTBD and mScarlet-tagged Gephyrin or GFP nanobodies.

The TRIM-Away method of antibody-based protein degradation^2,33,41,42^ can be coupled with GFP nanobodies to degrade GFP-tagged endogenous proteins^33^. Our high-resolution live imaging data showed that GFP nanobodies also efficiently recognize exogenously expressed mClover3-tagged proteins in mouse oocytes (Extended Data Fig. 4a). We thus predicted that GFP nanobody-coupled TRIM-Away of REC8 cohesin in REC8−FKBP12^F36V^−mClover3 mouse eggs could be used to induce aging-like premature separation of sister chromatids (Fig. 3a). To test this, we first microinjected exogenous TRIM21-expressing metaphase II stage eggs from engineered mice with mRNAs encoding GFP^43^ or Gephyrin^44^ (control) nanobodies and then visualized chromatid dynamics at high spatiotemporal resolution. Analyses of chromosome dynamics in these experiments showed that GFP nanobody-mediated TRIM-Away of endogenous REC8 accelerated premature separation and scattering of sister chromatids (Fig. 3b–e and Supplementary Videos 7 and 8). In addition, we observed up to four prematurely separated chromatids 1.5 hours after REC8 degradation (Fig. 3c). These quantitative analyses confirmed that engineered REC8−FKBP12^F36V^−mClover3 mice offer multiple opportunities to rapidly generate aging-like aneuploidies in young eggs via chemical or genetic manipulation approaches.

**Fig. 4:**
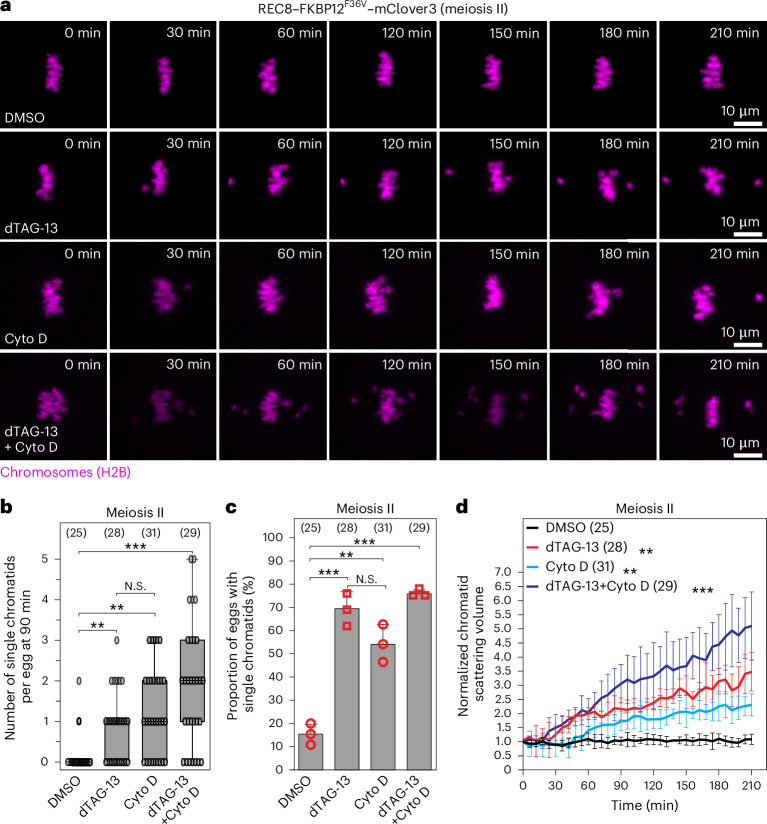
Premature sister chromatid separation arising from PROTAC-mediated REC8 cohesin degradation is accelerated by actin cytoskeleton disruption. **a**, Images from representative time-lapse movies of sister chromatids (H2B−mScarlet) in DMSO-treated, dTAG-13-treated, Cytochalasin D (CytoD)-treated or dTAG-13-treated and CytoD-treated eggs matured in vitro from REC8−FKBP12^F36V^−mClover3 oocytes. *t* = 0 minutes denotes the start of live imaging experiment. **b**, Quantification of the number of single chromatids per egg at 90 minutes of live imaging in DMSO-treated, dTAG-13-treated, CytoD-treated or dTAG-13-treated and CytoD-treated eggs matured in vitro from REC8−FKBP12^F36V^−mClover3 oocytes. **c**, Quantification of the proportion of cells containing single chromatids in DMSO-treated, dTAG13-treated, CytoD-treated or dTAG-13-treated and CytoD-treated eggs matured in vitro from REC8−FKBP12^F36V^−mClover3 oocytes. **d**, Measurements of chromatid scattering volumes from time-lapse movies of DMSO-treated, dTAG-13-treated, CytoD-treated or dTAG-13-treated and CytoD-treated eggs matured in vitro from REC8−FKBP12^F36V^−mClover3 oocytes. Lines represent mean values, and error bars indicate s.e.m. Data are from three independent experiments. Statistical significance was assessed using Student’s *t*-test (**b**,**c**) or two-way ANOVA (**d**). The number of analyzed oocytes is provided in brackets and italics. N.S., non-significant values. Source data

We previously identified that female reproductive aging is accompanied by meiotic spindle-specific disruption of the actin cytoskeleton, which predisposes mammalian eggs to premature chromatid separation^2^. To confirm that our new experimental system of female reproductive age-related aneuploidy recapitulates this new cytoskeletal function, we first treated metaphase II-arrested eggs with dTAG-13 to reduce chromosome cohesion and then disrupted F-actin with Cytochalasin D^2,36^. Consistent with our previous finding^2^, quantification of chromatid scattering volume in our high-spatiotemporal-resolution three-dimensional live imaging assays of chromosome dynamics^2,36^ revealed that F-actin disruption alone caused gradual splitting of sister chromatids in reproductively young REC8−FKBP12^F36V^−mClover3 females (Fig. 4a–d and Supplementary Videos 9 and 10), with an average of one single chromatid evident 1.5 hours after drug addition (Fig. 4b). Confirming our initial data, dTAG-13-mediated cohesion disruption caused significant chromatid separation and scattering (Fig. 4a–c and Supplementary Video 11), with up to three single chromatids present on the spindle after 1.5 hours of PROTAC treatment (Fig. 4b). Validating that this experimental system indeed recapitulates aneuploidies associated with female reproductive age-related F-actin dysfunction, Cytochalasin D addition accelerated the scattering of prematurely separated sister chromatids in dTAG-13-treated eggs containing reduced cohesion (Fig. 4d and Supplementary Video 12).

**Fig. 5:**
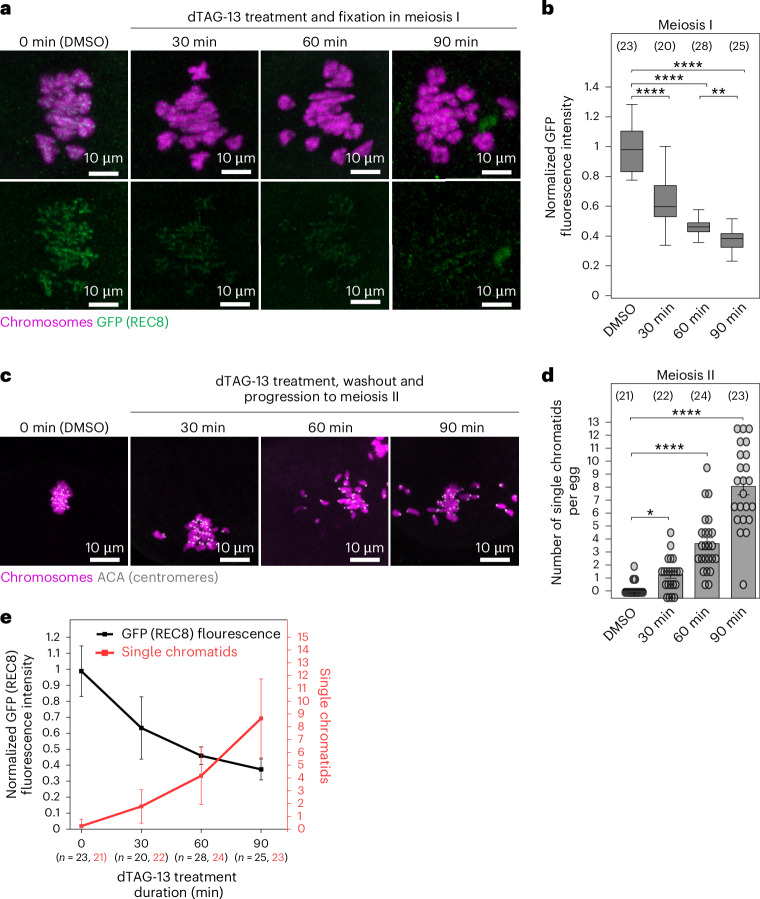
Partial REC8 degradation reveals a threshold-based model of cohesion loss in oocytes. **a**, Representative confocal images of REC8 (GFP antibody) and chromosomes (Hoechst) in metaphase I-stage REC8−FKBP12^F36V^−mClover3 oocytes treated with DMSO (0 minutes) or dTAG-13 for 30 minutes, 60 minutes or 90 minutes. **b**, Quantification of normalized REC8 fluorescence intensity in metaphase I oocytes treated with dTAG-13 for the indicated durations, fixed immediately after treatment. Fluorescence was measured on chromosomes and normalized to DMSO control. **c**, Representative confocal images of metaphase II chromosomes (Hoechst) and centromeres (ACA) in oocytes treated with dTAG-13 for the indicated durations during metaphase I, followed by washout and progression into meiosis II. **d**, Quantification of the number of prematurely separated sister chromatids per metaphase II egg after the treatment and washout scheme in **c**. **e**, Integrated analysis of the data from **b** and **d** showing the relationship between REC8 fluorescence intensity and the number of prematurely separated sister chromatids per egg. REC8 levels were measured in metaphase I oocytes immediately after treatment, and corresponding chromatid separation was quantified in metaphase II eggs after washout and meiotic progression. Chromatid separation remained minimal until REC8 levels dropped below approximately 50% of control, beyond which separation increased sharply, supporting a threshold-based model of cohesion loss. Data are from three independent experiments. Statistical comparisons were performed using Student’s *t*-test (**b**,**d**). The number of analyzed oocytes is indicated in brackets and italics. Source data

A central unresolved question in reproductive biology is why the incidence of egg aneuploidy rises exponentially at advanced maternal ages. One possibility is that cohesion loss is gradual over time, but errors remain rare until cohesion falls below a critical threshold, triggering a steep rise in chromosomal mis-segregation. Alternatively, cohesion weakening alone may be insufficient to cause widespread aneuploidy and, instead, must be compounded by additional age-associated defects to drive the exponential increase in errors. To distinguish between these possibilities, we treated metaphase I-stage oocytes with dTAG-13 for 0 minutes (DMSO control) to 90 minutes, fixed a subset of cells at each timepoint to quantify REC8 fluorescence intensity as a measure of cohesion levels (Fig. 5a,b) and allowed the remaining oocytes to proceed through anaphase I after dTAG-13 washout. We then assessed premature sister chromatid separation in the resulting metaphase II eggs (Fig. 5c,d). We found that modest reductions in REC8, down to approximately 60−70% of initial fluorescence intensity, had little effect on chromatid cohesion, with most eggs containing 0−2 prematurely separated chromatids. However, once REC8 levels declined below approximately 50%, the number of separated chromatids per egg rose sharply, reaching an average of four chromatids by 60 minutes and eight chromatids by 90 minutes of dTAG-13 treatment. This steep increase in the number of premature separation events supports a threshold-based model in which partial cohesion loss is tolerated until a critical level is crossed, beyond which meiotic chromosome integrity rapidly deteriorates. However, our previous work showed that actin disassembly exacerbates premature chromatid separation in aged eggs with weakened cohesion and, notably, is sufficient to induce these defects even in young oocytes with presumably intact cohesion^2^, underscoring its independent role in maintaining chromosome segregation fidelity. Collectively, these findings suggest that, whereas cohesion loss creates a latent vulnerability, the characteristic exponential rise in oocyte aneuploidy likely emerges from the synergistic failure of multiple pathways that safeguard chromosome segregation during female reproductive aging.

**Extended Data Fig. 5:**
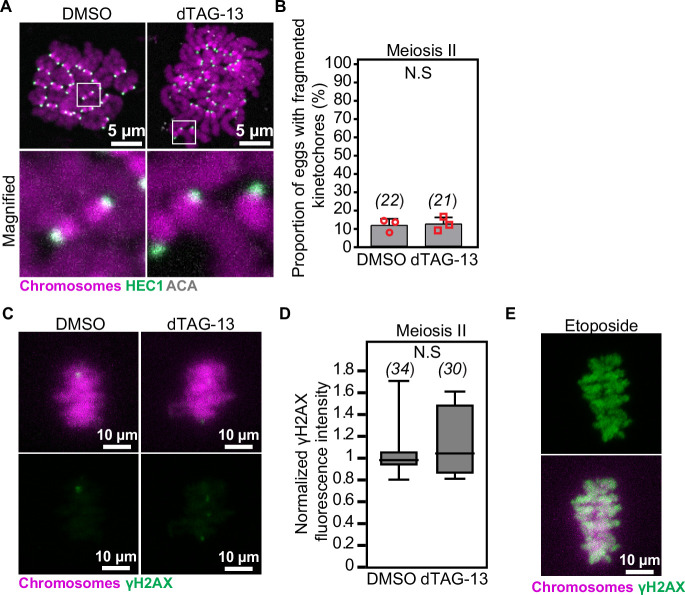
Acute REC8 degradation does not induce kinetochore fragmentation or DNA damage in young oocytes. (**A**) Representative confocal images of metaphase II-arrested REC8-FKBP12^F36V^-mClover3 eggs treated with DMSO or dTAG-13 and immunostained with ACA (centromere marker) and HEC1 (kinetochore protein). Insets show magnified views of kinetochores. (**B**) Quantification of the proportion of oocytes exhibiting fragmented kinetochores after treatment. No significant increase in kinetochore fragmentation was observed in dTAG-13–treated REC8-FKBP12^F36V^-mClover3 eggs compared to controls. (**C**) Representative images of γH2AX immunostaining in REC8-FKBP12^F36V^-mClover3 metaphase II eggs treated with DMSO or dTAG-13, showing comparable levels of DNA damage marker signal. (**D**) Quantification of normalized γH2AX fluorescence intensity. No significant increase in γH2AX signal was detected in dTAG-13-treated REC8-FKBP12^F36V^-mClover3 eggs. (**E**) Positive control: Representative γH2AX staining of wild-type metaphase II eggs treated with etoposide, a topoisomerase II inhibitor known to induce DNA damage, confirming assay sensitivity. Data are from three independent experiments. Statistical comparisons were performed using Student’s t-test. The number of analyzed oocytes is indicated in brackets and italics. Source data

To determine whether acute depletion of REC8 cohesin in young oocytes is sufficient to cause other phenotypes commonly associated with maternal aging, we examined two well-known markers of aged oocytes: kinetochore fragmentation in meiosis II and the accumulation of DNA damage. Kinetochore fragmentation is associated with the structural deterioration of centromeres in older oocytes^41^, whereas DNA damage, as detected by γH2AX staining, is another age-related feature thought to result from decreased genome stability^45–48^. We treated metaphase II-arrested REC8−FKBP12^F36V^−mClover3 oocytes with dTAG-13 to induce rapid cohesion weakening and assessed kinetochore structure using anti-centromere antibodies (ACA) and HEC1 immunostaining. Quantification of kinetochore morphology showed no significant increase in fragmentation after dTAG-13 treatment compared to DMSO controls (Extended Data Fig. 5a,b). Similarly, γH2AX fluorescence levels were similar between control and treated oocytes, indicating that REC8 degradation does not cause detectable DNA damage in young eggs (Extended Data Fig. 5c,d). As a positive control, we treated metaphase II-stage eggs with the topoisomerase II inhibitor etoposide^49,50^, which strongly induced γH2AX accumulation (Extended Data Fig. 5e), confirming the sensitivity of our detection method. The absence of these phenotypes in our system highlights its usefulness for isolating specific aging-related perturbations. Notably, because centromeric cohesion weakening underlies premature sister chromatid separation, a hallmark of oocyte aging, we conclude that our system accurately mimics this core feature of reproductive aging while preserving the broader cellular environment of a young oocyte.

**Fig. 6:**
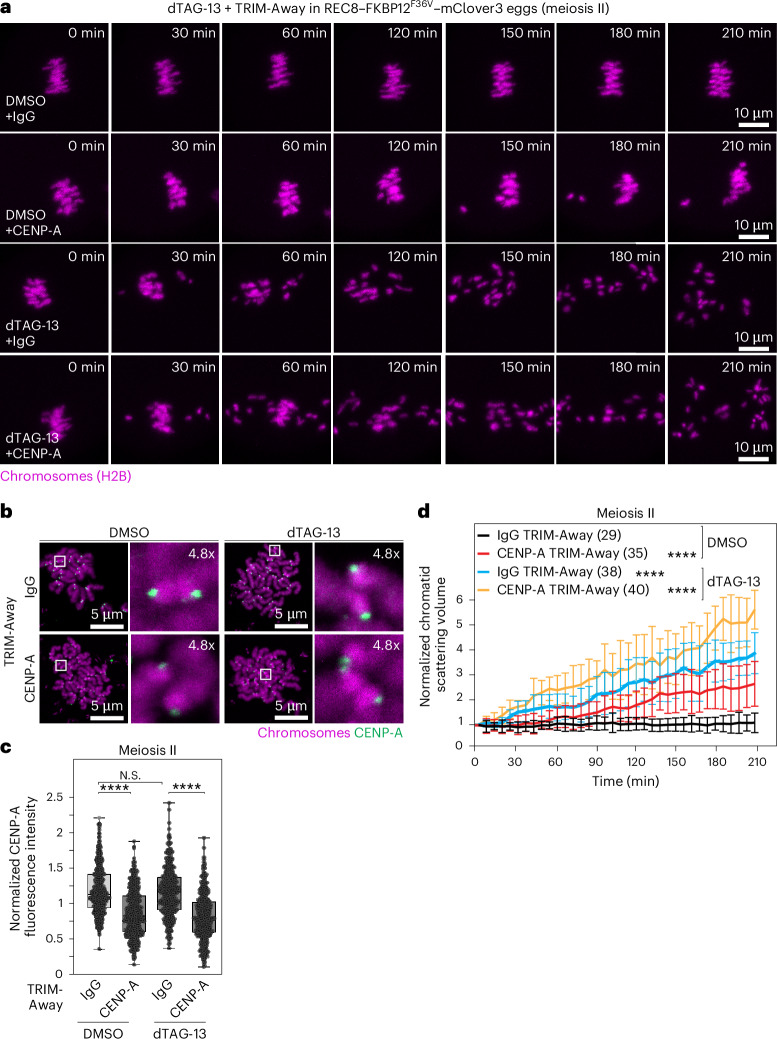
Aging-like CENP-A depletion triggers premature separation of sister chromatids independently of cohesion loss. **a**, Images from representative time-lapse movies of sister chromatids (H2B−mScarlet) in DMSO-treated and IgG TRIM-Away, DMSO-treated and CENP-A TRIM-Away, dTAG-13-treated and IgG TRIM-Away or dTAG-13-treated and CENP-A TRIM-Away eggs from REC8−FKBP12^F36V^−mClover3 mice. *t* = 0 minutes denotes the start of live imaging experiment. **b**, Representative maximum intensity projected immunofluorescence images of CENP-A and chromosomes in metaphase II chromosomal spreads of DMSO-treated and IgG TRIM-Away, DMSO-treated and CENP-A TRIM-Away, dTAG-13-treated and IgG TRIM-Away or dTAG-13-treated and CENP-A TRIM-Away eggs from REC8−FKBP12^F36V^−mClover3 mice. Boxes mark regions of interest that are 4.8× magnified. **c**, Quantification of CENP-A fluorescence intensities in metaphase II chromosomal spreads of DMSO-treated and IgG TRIM-Away (295 centromeres), DMSO-treated and CENP-A TRIM-Away (535 centromeres), dTAG-13-treated and IgG TRIM-Away (347 centromeres) and dTAG-13-treated and CENP-A TRIM-Away (465 centromeres) eggs from REC8−FKBP12^F36V^−mClover3 mice. **d**, Measurements of chromatid scattering volumes from time-lapse movies of DMSO-treated and IgG TRIM-Away, DMSO-treated and CENP-A TRIM-Away, dTAG-13-treated and IgG TRIM-Away or dTAG-13-treated and CENP-A TRIM-Away eggs from REC8−FKBP12^F36V^−mClover3 mice. Lines represent mean values, and error bars indicate s.e.m. Data are from three independent experiments. Statistical significance was assessed using two-way ANOVA (**c**,**d**). The number of analyzed oocytes is provided in brackets and italics. Source data

**Extended Data Fig. 6:**
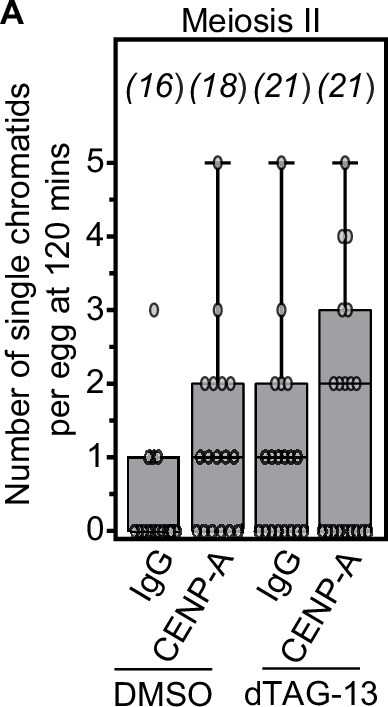
CENP-A TRIM-Away increases the number of prematurely separated sister chromatids in dTAG-13-mediated cohesion degradation. (**A**) Quantification of the number of single chromatids per egg at 120 min of live imaging in DMSO-treated and IgG TRIM-Away, DMSO-treated and CENP-A TRIM-Away, dTAG-13-treated and IgG TRIM-Away or dTAG-13-treated and CENP-A TRIM-Away eggs from REC8-FKBP12^F36V^-mClover3 mice. Source data

A small-molecule-based tool that directly manipulates chromosome cohesion holds strong promise for the rapid screening and discovery of proteins whose age-related disruption can predispose eggs to aneuploidy, independently of cohesion loss. We thus tested whether our PROTAC-based experimental system of oocyte aging can be applied to discover factors associated with the remarkable rise in egg aneuploidy at advanced reproductive ages^4–6^. The histone H3 variant CENP-A^51^ is required for proper centromere function in oocytes, fertility and transmission of the genome to the next generation^52,53^. Reproductive age-related depletion of CENP-A and other centromeric components was recently proposed to underlie kinetochore dysfunction and chromosome segregation errors in aged oocytes^54^. However, it is unknown whether CENP-A loss can trigger the sharp increase in the incidence of egg aneuploidy observed during maternal aging^4–6^. We addressed this by first treating exogenous TRIM21-expressing REC8−FKBP12^F36V^−mClover3 metaphase II-stage eggs for 3 hours with DMSO (control) or dTAG-13. We then performed control or CENP-A TRIM-Away by microinjecting mouse IgG or CENP-A heavy chain antibodies into DMSO-treated or dTAG-13-treated eggs. Immunofluorescence microscopy of metaphase chromosome spreads showed that CENP-A protein abundance was successfully reduced by approximately 30% after TRIM-Away (Fig. 6b,c), a value similar to CENP-A depletion levels in naturally aged eggs^54^. dTAG-13 treatment of control IgG TRIM-Away eggs induced modest separation and scattering of sister chromatids at 2 hours of treatment (Fig. 6a,d, Extended Data Fig. 6a and Supplementary Videos 13 and 14). TRIM-Away-mediated aging-like degradation of CENP-A in DMSO-treated control eggs led to chromatid scattering events similar to dTAG-13-mediated partial removal of cohesion (Fig. 6a,d, Extended Data Fig. 6a and Supplementary Video 15). Because CENP-A is critical for the identity and function of centromeres that also serve as sole regions of meiosis II chromosome cohesion^5,17,18,20,40^, failure to maintain centromeric cohesion in CENP-A TRIM-Away eggs could explain this observation. Notably, when we combined REC8 dTAG-13 and CENP-A TRIM-Away perturbations, we observed a sustained and significantly amplified increase in chromatid scattering volume over time compared to either single perturbation (Fig. 6a,d, Extended Data Fig. 6a and Supplementary Video 16). These results support a model in which age-related defects in distinct cohesion mechanisms act cooperatively to destabilize chromosome structure, and they demonstrate the strength of our experimental system in dissecting such interactions with temporal and quantitative precision.

## Discussion

In this study, we developed a versatile experimental system for induction of aging-like chromosomal phenotypes in young eggs. By overcoming the need to age mice or purchase aged animals, this drug-based cohesion manipulation system provides a fast and cost-effective approach to discover the causes of reproductive age-related egg aneuploidy.

Although previous studies introduced valuable tools to manipulate cohesion, including systems based on TEV-protease-cleavable REC8 and separase activation^40,55^, these approaches rely on the injection of recombinant proteins or irreversible enzymatic cleavage, which limits their temporal precision and adaptability. Such systems are not easily tunable, and they cannot precisely control the duration of perturbation, which is essential for modeling the progressive and threshold-based nature of cohesion loss during aging. By contrast, our system enables acute and titratable degradation of endogenous REC8 with minute-scale temporal resolution, and, critically, the response can be terminated on demand by simple washout of dTAG-13, as demonstrated in our experiments where metaphase I treatment induces premature sister chromatid separation detectable in metaphase II. This level of control is not possible with irreversible or constitutive systems.

We anticipate that innovative live imaging assays of REC8 protein dynamics enabled by this experimental system will also provide new opportunities to probe the mechanisms of chromosome cohesion in female meiosis. Next steps will include combining this approach with libraries of small molecules, PROTACs and clinical compounds for unbiased identification and mechanistic studies of new meiotic fidelity proteins. In the future, clinical manipulation of meiotic processes uncovered by this approach could accelerate next-generation fertility treatments that reduce the incidence of aneuploidy in fertilizable eggs.

## Methods

### Mouse strains and husbandry

All protocols involving mice were approved by the Yale University Institutional Animal Care and Use Committee (approval number 2021-20408). Mice were housed under specific pathogen-free conditions with a 12-hour light/dark cycle. The mouse strains used in this study included REC8−FKBP12^F36V^−mClover3 and wild-type C57BL/6J (The Jackson Laboratory).

### CRISPR−Cas generation of REC8−FKBP12^F36V^−mClover3 mice

The REC8−FKBP12^F36V^−mClover3 mouse model was generated via CRISPR−Cas9-mediated genome editing^56–58^. Potential Cas9 target guide (protospacer) sequences near the REC8 translation termination site were screened using the online tool CRISPOR (http://crispor.tefor.net (ref. ^59^)), and candidates were selected. crRNAs containing the chosen sequences were synthesized (Integrated DNA Technologies (IDT)). crRNA/tracrRNA/Cas9 ribonucleoproteins (RNPs) were complexed and tested for activity by zygote electroporation, incubation of embryos to blastocyst stage and genotype scoring of indel creation at the target sites. A gRNA that demonstrated high activity and that directed Cas9 cleavage to the translation termination codon, 5′-GGTTTAATGAACTTGCTCAG, was selected for creating the knockin allele. Accordingly, a 1.5-kb long single-stranded DNA (lssDNA) recombination template containing the FKBP12^F36V^−mClover3 element was synthesized (IDT). The injection mix of gRNA/Cas9 RNP + lssDNA was microinjected into the pronuclei of C57Bl/6J zygotes^58^. Embryos were transferred to the oviducts of pseudo-pregnant CD-1 foster females using standard techniques^60^. Genotype screening of tissue biopsies from founder pups was performed by polymerase chain reaction (PCR) amplification and Sanger sequencing to verify the knockin allele. Germline transmission of the correctly targeted allele was confirmed by breeding and sequence analysis. To maintain genetic robustness, the REC8−FKBP12^F36V^−mClover3 colony was outbred to C57BL/6J mice every five generations. After the establishment of homozygosity, this strain was maintained as an independent line for subsequent experiments.

### Genotyping of REC8−FKBP12^F36V^−mClover3 mice

Genotyping was performed using DNA extracted from ear punch biopsies. DNA was amplified using KAPA HiFi HotStart ReadyMix (Roche, 07958935001) according to the manufacturer’s instructions. PCR primers specific for REC8 (Supplementary Table 1; BM729-BM735) were used to distinguish between alleles. The expected product sizes were 550 bp for the wild-type allele and 1,700 bp for the REC8−FKBP12^F36V^−mClover3 allele.

### Mouse oocyte isolation, maturation, culture and microinjection

Oocytes were collected from ovaries of 6–8-week-old REC8−FKBP12^F36V^−mClover3 female mice. Isolated oocytes were matured in vitro and microinjected with 6–8 pl of in vitro transcribed mRNA as previously described^36^.

### Generation of in vitro mRNA transcription constructs and mRNA synthesis

Oligonucleotide sequences used for plasmid construction are provided in Supplementary Table 1. All synthesized gene sequences were obtained from Twist Bioscience. To generate pGEM−H2B−mScarlet, the coding sequence of mouse H2B (obtained by gene synthesis) was amplified with KS109 and KS110 and transferred into pGEM−mScarlet backbone that was linearized with EcoRI and XhoI. pGEM−MAP4−MTBD−mClover was generated by Gibson assembly (New England Biolabs) of KS111 and KS112 amplified synthetic mClover3 sequence into the AflII and NcoI site of pGEM−EGFP−MAP4−MTBD^2^. pGEM−mNeonGreen−MAP4−MTBD was constructed by Gibson assembly of KS158 and KS159 amplified synthetic mNeonGreen sequence into pGEM−MAP4−MTBD−mClover3 that was linearized with KS156 and KS157. Generation of pGEM−SNAP−TRIM21 construct was described previously^2^. pGEM−VHHGFP4−hIgG1−Fc1 plasmid was constructed using Gibson assembly. A PCR fragment amplified with primers BM343 and BM344 from the pcDNA3−Nslmb−VHHGFP4 (ref. ^43^) (Addgene, 35579) plasmid was inserted into the HindIII–EcoRI site of the pGEM−hIgG1−Fc1 vector^3^. pGEM−VHHGFP4−mScarlet was generated by linearizing pGEM−HE^61^ with BM715 and BM811 and transferring into it using Gibson assembly BM812 and BM813 flanked synthesized coding sequence of VHHGFP4. Gephyrin nanobody sequence^44^ was obtained by gene synthesis. pGEM−GephyrinVHH−hlgG1−Fc1 was generated by transferring JL7 and JL8 amplified coding sequence of Gephyrin nanobody into the HindIII–EcoRI site of pGEM−hIgG1−Fc1. pGEM−GephyrinVHH−mScarlet was generated by linearizing pGEM−HE with JL9 and JL10 and transferring into it by Gibson assembly JL3 and JL4 flanked synthetic coding sequence of Gephyrin VHH. pGEM−mScarlet−NLS was constructed by amplifying the coding sequence of mScarlet−NLS (synthesized DNA) using primers BM432 and BM433 and transferring it into the HindIII site of pGEM−HE using Gibson assembly. pGEM−mClover3−NLS was generated using Gibson assembly to transfer BM432 and FG40 flanked mClover3 coding sequence into pGEM−mScarlet−NLS that was linearized with FG39 and BM494.

Capped mRNAs were synthesized using a T7 polymerase (Ambion, mMessage mMachine kit). mRNA concentrations were measured using a NanoDrop spectrophotometer (Thermo Fisher Scientific).

### Drug addition and fluorescence labeling experiments

Stock solutions of all small-molecule inhibitors were prepared in DMSO. To disrupt actin, oocytes were treated with Cytochalasin D (Merck, C8273-1MG) at a final concentration of 5 μg ml^−1^ in M2 medium for 1 hour. For rapid protein degradation experiments, eggs were exposed to 3 μM dTAG-13 (Merck, SML2601). All compounds were dissolved in DMSO (Merck, D2650-5X5ML) and diluted in M2 medium. In live imaging experiments, chromosomes were visualized by treating cells for 1 hour with 100 nM SiR-5-Hoechst dye diluted in M2 medium. DMSO was used as control samples at a final concentration of 0.003% in M2 medium.

### Western blotting

Protein samples were prepared by washing sperm in PBS and lysing in LDS sample buffer (Invitrogen, NuPAGE). Samples were agitated thoroughly and snap frozen at −80 °C until use. Before electrophoresis, samples were boiled at 95 °C for 10 minutes with reducing agent (NuPAGE). Proteins were separated on 4–12% Bis-Tris gels (Invitrogen, NuPAGE) and transferred to PVDF membranes (Millipore, Immobilon-P). Membranes were blocked with 3% BSA in PBS for 1 hour at room temperature. Primary antibody incubation was performed overnight at 4 °C in 5% (w/v) milk-PBST (PBS containing 0.05% (w/v) Tween 20) blocking buffer with GFP antibody (1:1,000; Novus Biologicals, NBP2-50059). Membranes were washed for 5 minutes three times with PBST and then incubated with goat anti-rabbit IgG StarBright Blue 700 secondary antibody (1:5,000, Bio-Rad) prepared in blocking buffer for 1 hour at room temperature. Actin-Rhodamine (Bio-Rad) was used as a loading control to detect actin. After three additional washes in PBST, fluorescence signals were detected using the ChemiDoc MP Imaging System (Bio-Rad) with auto-exposure mode.

### Fixation and immunostaining of mouse oocytes and eggs

Oocytes were fixed in a solution containing 100 mM HEPES, 10 mM MgSO_4_, 50 mM EGTA, 0.5% Triton X-100 (v/v) and 2% formaldehyde (v/v) at 37 °C for 30 minutes. Fixed cells were blocked overnight at 4 °C in PBT (PBS containing 0.3% Triton X-100 (v/v)) and 3% BSA (w/v). Primary antibodies anti-GFP (1:100 dilution; Roche, 11814460001), anti-phospho-histone H2A.X (Ser139) (1:250 dilution; Merck, 05-636) and human anti-centromere antibody (1:1,000 dilution; Antibodies Incorporated, 15-234) were incubated overnight at 4 °C. Cells were washed and incubated with Alexa Fluor 488-labeled anti-mouse secondary antibody (1:500; Molecular Probes), Alexa Fluor 647-labeled anti-human antibody (1:2,000 dilution; Molecular Probes) and Hoechst 33342 (5 μg ml^−1^; Molecular Probes) for 1 hour at room temperature.

### Metaphase chromosomal spreading, fixation and immunostaining

Metaphase II-arrested eggs were prepared for chromosomal spreading by removing the zona pellucida using Tyrode’s acid solution (Sigma-Aldrich, T1788). Eggs were then thoroughly washed through droplets of fresh M2 medium and recovered by incubating for at least 10 minutes at 37 °C. For chromosomal spreading, single cells were placed on a slide in a fixative solution containing 1% formaldehyde (v/v), 0.15% Triton X-100 (v/v) and 3% dithiothreitol (v/v), pH 9.2–9.4. The slides were dried slowly in a humid chamber for several hours and then blocked in PBS containing 3% BSA (w/v) for 1 hour. Spreads were then incubated with λ-phosphatase (New England Biolabs, P0753S) at 30 °C for 2 hours. Immunostaining was performed by washing out the blocking solution with PBS, followed by incubation with primary antibodies overnight at 4 °C and secondary antibodies for 2 hours at room temperature. Primary antibodies were human anti-centromere (1:1,000 dilution; Antibodies Incorporated, 15-234), CENP-A (1:200 dilution; Cell Signaling Technology, C51A7) and HEC1 (1:500 dilution; Santa Cruz Biotechnology, sc-135934). Secondary antibodies and stains used were Alexa Fluor 488-labeled anti-rabbit (1:2,000 dilution; Molecular Probes), Alexa Fluor 647-labeled anti-human (1:2,000 dilution; Molecular Probes), Alexa Fluor 488-conjugated anti-rabbit (1:500; Molecular Probes) and Hoechst 33342 (5 μg ml^−1^; Molecular Probes). Slides were washed and mounted with VECTASHIELD Antifade Mounting Medium (2B Scientific, H-1000-10) under 22 × 22-mm glass coverslips (Epredia, 22×22-1-002G), which were sealed with nail varnish.

### TRIM-Away-mediated protein degradation in dTAG-13-treated mouse eggs

For nanobody-mediated TRIM-Away of Gephyrin or GFP proteins in dTAG-13-treated eggs, metaphase II-arrested eggs expressing TRIM21 were microinjected in M2 medium with 2–3 pl of GFP or Gephyrin nanobody-encoding mRNAs.

In CENP-A degradation TRIM-Away experiments, TRIM21-expressing metaphase II-arrested eggs were incubated in 3 μM DMSO or dTAG-13 for 1.5 hours before microinjection of control rabbit-IgG (R&D Systems, A-105-C) or CENP-A (Cell Signaling Technology, C51A7) antibodies. Successful microinjection was confirmed by co-microinjection of fluorescent Dextran Texas Red (1:100 dilution; Invitrogen, D1829) in 0.05% (v/v) NP-40-PBS. All TRIM-Away microinjections and successive live imaging experiments were performed in M2 medium containing DMSO or dTAG

### In vitro fertilization and culturing of mouse embryos

Female REC8−FKBP12^F36V^−mClover3 mice (6–8 weeks old) were superovulated by intraperitoneal injection of 5 IU or 7.5 IU of pregnant mare serum gonadotropin (PMSG; ProSpec, HOR-272), followed 48 hours later by an injection of 5 IU or 7.5 IU of human chorionic gonadotropin (hCG; Sigma-Aldrich, CG-10). Thirteen hours after hCG injection, metaphase II-arrested eggs were collected from the ampullae of the oviducts. Cumulus cells were removed with a brief treatment using hyaluronidase (300 μg ml^−1^ in M2 medium; Sigma-Aldrich, H4272), and zona pellucida–intact eggs were then thoroughly washed in M2 medium.

For chemical degradation experiments, eggs were cultured in M2 medium supplemented with either DMSO or 3 μM dTAG-13 at 37 °C for 1 hour.

Sperm was collected from the cauda epididymides of >12-week-old REC8−FKBP12^F36V^−mClover3 males and capacitated for 2 hours in human tubal fluid (HTF) medium (Millipore, MR-070-D) supplemented with 2 mM hypotaurine (Sigma-Aldrich, H1384) at 37 °C and 5% CO_2_. In vitro fertilization was performed by adding 2–5 μl of capacitated sperm into 40-μl HTF droplets with the treated eggs. After 2 hours of co-incubation, fertilized zygotes were washed through fresh KSOM medium (MR-101) and cultured in 30-μl KSOM droplets under mineral oil at 37 °C in a humidified atmosphere containing 5% CO_2_.

### High-resolution live-cell imaging

Confocal time-lapse images of mouse oocyte and metaphase II-arrested eggs were acquired using a Zeiss LSM 800 Airyscan or LSM 900 Airyscan 2 microscope equipped with a ×40 C-Apochromat 1.2 numerical aperture water immersion objective and an environmental chamber maintained at 37 °C. Image acquisition was controlled with ZEN2 software (Zeiss) and conducted at a temporal resolution of 6 minutes. REC8−mClover3 was imaged on a Leica STELLARIS 5 confocal laser scanning microscope equipped with an environmental incubator box and a ×40 C-Apochromat 1.2 numerical aperture water immersion objective and conducted at a temporal resolution of 3 minutes. *z* stacks were captured with a thickness of approximately 40 μm, with confocal sections spaced 1.5 μm apart. Oocytes were imaged in M2 medium under mineral oil, as described previously^36^.

### Immunofluorescence microscopy

Confocal immunofluorescence imaging was performed using a Zeiss LSM 800 or LSM 900 confocal microscope with a ×40 C-Apochromat 1.2 numerical aperture water immersion objective. *z* stacks were acquired with a thickness of 2.0 μm, with 0.5-μm confocal sections.

For GFP immunofluorescence imaging, images were acquired in 1-μm steps over a 4-μm range at the midplane of meiotic chromosomes using the Airyscan module on Zeiss LSM 800 or Airyscan 2 module LSM 900 microscopes. Post-acquisition super-resolution images were generated through three-dimensional Airyscan processing using ZEN2 software (Zeiss). Eggs were imaged in M2 medium under mineral oil, as described previously^36^.

### Measurement of fluorescence intensity in metaphase chromosome spreading assays

Mean fluorescence intensity of REC8 on metaphase I chromosomes was measured in ImageJ by manually tracing regions of interest around maximum intensity projected images of chromosome spreads. Mean fluorescence intensity of CENP-A on metaphase II sister chromatids was measured in ImageJ by manually tracing centromeric regions of DNA in maximum intensity projected images of chromosome spreads.

### Three-dimensional surface reconstruction and measurement of chromatid scattering volume

Chromatid surfaces were reconstructed in three dimensions using Imaris (Bitplane) from high-resolution time-lapse movies of H2B−mScarlet. Chromatid scattering volume was quantified using object-oriented bounding box analysis in Imaris, identifying the minimal cuboid volume enclosing all chromatids at each timepoint. For each egg, the scattering volume was normalized by dividing the bounding box volume at each timepoint by the volume at the start of the live imaging experiment (*t* = 0 minutes). This normalization enabled direct comparison of chromatid dynamics across samples.

### Statistical data analysis

All statistical analyses were conducted using GraphPad Prism (GraphPad Software) or OriginPro (OriginLab). Statistical box plots represent median (line), mean (small square), 5th and 95th percentiles (whiskers) and 25th and 75th percentiles (box enclosing 50% of the data). Normalizations were performed by dividing individual data points in control and experimental groups by the average value of control group data points. Differences between two groups were analyzed using Student’s *t*-test, whereas comparisons among more than two groups were assessed using two-way ANOVA. Statistical significance is denoted as *P < 0.05, **P < 0.005 and ***P < 0.0005. Non-significant values are indicated as ‘NS’.

### Reporting summary

Further information on research design is available in the Nature Portfolio Reporting Summary linked to this article.

## Supplementary information

Supplementary Information. Oligonucleotide sequences used in this study.

Reporting Summary

Supplementary Video 1 Navigation through 1 µm apart confocal sections of REC8 (GFP antibody, grey) and homologous chromosomes (magenta) in a metaphase I-stage mouse oocyte.

Supplementary Video 2 High-resolution time lapse movie showing the selective removal of REC8 from chromosome arms and its retention at centromeric regions during anaphase I in a REC8-FKBP12F36V-mClover3 mouse oocyte.

Supplementary Video 3 Time lapse movie of chromosome alignment and segregation during meiosis I in a wild-type mouse oocyte. Microtubules (grey) are labeled with mNeonGreen-MAP4-MTBD and chromosomes (magenta) are labeled with H2B-mScarlet.

Supplementary Video 4 Time lapse movie of chromosome alignment and segregation during meiosis I in a REC8-FKBP12F36V-mClover3 mouse oocyte. Microtubules (grey) are labeled with mNeonGreen-MAP4-MTBD and chromosomes (magenta) are labeled with H2B-mScarlet.

Supplementary Video 5 Time lapse movie of chromatids (H2B-mScarlet, grey) in a DMSO-treated (dTAG-13 control), metaphase II-arrested REC8-FKBP12F36V-mClover3 egg.

Supplementary Video 6 Time lapse movie of chromatids (H2B-mScarlet, grey) in a dTAG-13-treated, metaphase II-arrested REC8-FKBP12F36V-mClover3 egg.

Supplementary Video 7 Time lapse movie of chromatids (H2B-mScarlet, grey) in a Gephyrin TRIM-Away, metaphase II-arrested REC8-FKBP12F36V-mClover3 egg.

Supplementary Video 8 Time lapse movie of chromatids (H2B-mScarlet, grey) in a GFP TRIM-Away, metaphase II-arrested REC8-FKBP12F36V-mClover3 egg.

Supplementary Video 9 Time lapse movie of chromatids (H2B-mScarlet, grey) in a DMSO-treated (CytoD control), metaphase II-arrested REC8-FKBP12F36V-mClover3 egg.

Supplementary Video 10 Time lapse movie of chromatids (H2B-mScarlet, grey) in a CytoD-treated, metaphase II-arrested REC8-FKBP12F36V-mClover3 egg.

Supplementary Video 11 Time lapse movie of chromatids (H2B-mScarlet, grey) in a dTAG-13-treated, metaphase II-arrested REC8-FKBP12F36V-mClover3 egg.

Supplementary Video 12 Time lapse movie of chromatids (H2B-mScarlet, grey) in a CytoD- and dTAG-13-treated, metaphase II-arrested REC8-FKBP12F36V-mClover3 egg.

Supplementary Video 13 Time lapse movie of chromatids (H2B-mScarlet, grey) in a DMSO-treated and IgG TRIM-Away metaphase II-arrested REC8-FKBP12F36V-mClover3 egg.

Supplementary Video 14 Time lapse movie of chromatids (H2B-mScarlet, grey) in a DMSO-treated and CENP-A TRIM-Away metaphase II-arrested REC8-FKBP12F36V-mClover3 egg.

Supplementary Video 15 Time lapse movie of chromatids (H2B-mScarlet, grey) in a dTAG-13-treated and IgG TRIM-Away metaphase II-arrested REC8-FKBP12F36V-mClover3 egg.

Supplementary Video 16 Time lapse movie of chromatids (H2B-mScarlet, grey) in a dTAG-13-treated and CENP-A TRIM-Away metaphase II-arrested REC8-FKBP12F36V-mClover3 egg.

## Source data

Source Data Fig. 1 Statistical Source Data.

Source Data Fig. 2 Statistical Source Data.

Source Data Fig. 3 Statistical Source Data.

Source Data Fig. 4 Statistical Source Data.

Source Data Fig. 5 Statistical Source Data.

Source Data Fig. 6 Statistical Source Data.

Source Data Extended Data Fig. 1 Unprocessed gel.

Source Data Extended Data Fig. 2 Statistical Source Data.

Source Data Extended Data Fig. 3 Unprocessed western blots.

Source Data Extended Data Fig. 5 Statistical Source Data.

Source Data Extended Data Fig. 6 Statistical Source Data.

## Data Availability

All data are available in the main text or the supplementary materials. Plasmid constructs used in this study will be shared upon reasonable request and will be made publicly available through Addgene. REC8−FKBP12^F36V^−mClover3 mice are in the process of being deposited to The Jackson Laboratory for sharing with the wider research community (expected availability in 2026). Source Data files are available for this article.
