# Supplementary material for: A versatile cohesion manipulation system probes female reproductive age-related egg aneuploidy
Source: Nat Aging. 2025 Nov 3;5(11):2215–27. doi: 10.1038/s43587-025-00997-w (PMC12618256; doi:10.1038/s43587-025-00997-w)
Supplement: Supplementary file 1 — Oligonucleotide sequences used in this study. [file 43587_2025_997_MOESM1_ESM.pdf]

nature aging

Supplementary information

https://doi.org/10.1038/s43587-025-00997-w

A versatile cohesion manipulation system probes female reproductive age-related egg aneuploidy

In the format provided by the authors and unedited

Table S1. Oligonucleotide sequences used in this study.

Primer Name	Primer Sequence
BM729	TCGATGCCCTTCAGCTCGAT
BM730	CCACCCTCGTGACCACCT
BM732	ggctgctacagcacactcta
BM733	CCACCCCGGTGAACAGCT
BM734	gttatcccagctgacacccc
BM735	aaaggtggcctgtcttctgc
KS109	agcgctaccggtctcagatcATGCCAGAGCCAGCGAAG
KS110	tcccgggccgtcgactgcagaattCTTAGCGCTGGTGTACTTGG
KS156	gctgtacaagcttaaggagttcgtgaccgccgc
KS157	tgctcaccatggtggcgaccggtagcgc
KS158	ctagcgctaccggtcgccaccATGGTGAGCAAGGGCGAG
KS159	cggcggcggtcacgaactccttaagCTTGTACAGCTCGTCCATGC
KS111	ggtcgccaccatggtgagcaagggcgag
KS112	actccttaagcttgtacagctcgtccatgc
BM343	ggtctcagatctcgagctcaATGGATCAAGTCCAACTGGTG
BM344	tcagatctaaccatctgcagGCTGGAGACGGTGACCTG
JL7	ggtctcagatctcgagctcaATGGCCGAGGTGCAGCTG
JL8	tcagatctaaccatctgcagTGCGCCATGGTGATGGTG
BM715	CTTGTACAGCTCGTCCATG
BM811	tggacgagctgtacaagatcATGGCAGAACAAGGGGCC
BM812	aattcgaagcttgagctcgaCTAGATCACCTCGGCCGTTTG
BM813	GTGCAGGCAGAAGATGAAG
JL9	AGGAATTCGATATCAAGCTTC
JL10	AGCTCGAGATCTGAGACC
JL3	ccggtctcagatctcgagctATGGCCGAGGTGCAGCTG
JL4	aagcttgatatcgaattcctTGCGCCATGGTGATGGTG
BM432	ggtctcagatctcgagctcaATGGTGAGCAAGGGCGAG
BM433	ccgtcgactgcagaattcgaTTACACCTTCCGCTTTTTCTTAGG
FG39	CTGCAGGGGATGGTGAGC
BM494	TGAGCTCGAGATCTGAGAC
FG40	CACCTTCCGCTTTTTCTTAGGC
