# Supplementary material for: A versatile cohesion manipulation system probes female reproductive age-related egg aneuploidy
Source: Nat Aging. 2025 Nov 3;5(11):2215–27. doi: 10.1038/s43587-025-00997-w (PMC12618256; doi:10.1038/s43587-025-00997-w)
Supplement: Supplementary file 2 — Reporting Summary [file 43587_2025_997_MOESM2_ESM.pdf]

nature portfolio

Corresponding author(s): Binyam Mogessie

Last updated by author(s): Aug 15, 2025

Reporting Summary

Nature Portfolio wishes to improve the reproducibility of the work that we publish. This form provides structure for consistency and transparency in reporting. For further information on Nature Portfolio policies, see our Editorial Policies and the Editorial Policy Checklist.

Statistics

For all statistical analyses, confirm that the following items are present in the figure legend, table legend, main text, or Methods section.

n/a	Confirmed	
☐	☒	The exact sample size (*n*) for each experimental group/condition, given as a discrete number and unit of measurement
☐	☒	A statement on whether measurements were taken from distinct samples or whether the same sample was measured repeatedly
☐	☒	The statistical test(s) used AND whether they are one- or two-sided<br>*Only common tests should be described solely by name; describe more complex techniques in the Methods section.*
☒	☐	A description of all covariates tested
☒	☐	A description of any assumptions or corrections, such as tests of normality and adjustment for multiple comparisons
☐	☒	A full description of the statistical parameters including central tendency (e.g. means) or other basic estimates (e.g. regression coefficient) AND variation (e.g. standard deviation) or associated estimates of uncertainty (e.g. confidence intervals)
☒	☐	For null hypothesis testing, the test statistic (e.g. *F*, *t*, *r*) with confidence intervals, effect sizes, degrees of freedom and *P* value noted<br>*Give P values as exact values whenever suitable.*
☒	☐	For Bayesian analysis, information on the choice of priors and Markov chain Monte Carlo settings
☒	☐	For hierarchical and complex designs, identification of the appropriate level for tests and full reporting of outcomes
☒	☐	Estimates of effect sizes (e.g. Cohen's *d*, Pearson's *r*), indicating how they were calculated

Our web collection on statistics for biologists contains articles on many of the points above.

Software and code

Policy information about availability of computer code

Data collection	Zeiss ZEN 3.5 and Leica LASX 3.3 software were used for image acquisition on ZEISS LSM 800/900 and LEICA Stellaris microscopes
Data analysis	Imaris (Bitplane) version 9, Prism (graphpad) version 10 and Origin (OriginLab) 2023 software were used for image analyses and statistical analyses.

For manuscripts utilizing custom algorithms or software that are central to the research but not yet described in published literature, software must be made available to editors and reviewers. We strongly encourage code deposition in a community repository (e.g. GitHub). See the Nature Portfolio guidelines for submitting code & software for further information.

Data

Policy information about availability of data

All manuscripts must include a data availability statement. This statement should provide the following information, where applicable:

- Accession codes, unique identifiers, or web links for publicly available datasets
- A description of any restrictions on data availability
- For clinical datasets or third party data, please ensure that the statement adheres to our policy

All data are available in the main text or the supplementary materials. Plasmid constructs used in this study will be shared upon request and will be made publicly available through Addgene. REC8-FKBP12F36V-mClover3 mice are in the process of being deposited to Jackson Laboratories for sharing with the wider research community.

Research involving human participants, their data, or biological material

Policy information about studies with human participants or human data. See also policy information about sex, gender (identity/presentation), and sexual orientation and race, ethnicity and racism.

Reporting on sex and gender	*Use the terms sex (biological attribute) and gender (shaped by social and cultural circumstances) carefully in order to avoid confusing both terms. Indicate if findings apply to only one sex or gender; describe whether sex and gender were considered in study design; whether sex and/or gender was determined based on self-reporting or assigned and methods used. Provide in the source data disaggregated sex and gender data, where this information has been collected, and if consent has been obtained for sharing of individual-level data; provide overall numbers in this Reporting Summary. Please state if this information has not been collected. Report sex- and gender-based analyses where performed, justify reasons for lack of sex- and gender-based analysis.*
Reporting on race, ethnicity, or other socially relevant groupings	*Please specify the socially constructed or socially relevant categorization variable(s) used in your manuscript and explain why they were used. Please note that such variables should not be used as proxies for other socially constructed/relevant variables (for example, race or ethnicity should not be used as a proxy for socioeconomic status). Provide clear definitions of the relevant terms used, how they were provided (by the participants/respondents, the researchers, or third parties), and the method(s) used to classify people into the different categories (e.g. self-report, census or administrative data, social media data, etc.) Please provide details about how you controlled for confounding variables in your analyses.*
Population characteristics	*Describe the covariate-relevant population characteristics of the human research participants (e.g. age, genotypic information, past and current diagnosis and treatment categories). If you filled out the behavioural & social sciences study design questions and have nothing to add here, write "See above."*
Recruitment	*Describe how participants were recruited. Outline any potential self-selection bias or other biases that may be present and how these are likely to impact results.*
Ethics oversight	*Identify the organization(s) that approved the study protocol.*

Note that full information on the approval of the study protocol must also be provided in the manuscript.

Field-specific reporting

Please select the one below that is the best fit for your research. If you are not sure, read the appropriate sections before making your selection.

☒ Life sciences ☐ Behavioural & social sciences ☐ Ecological, evolutionary & environmental sciences

For a reference copy of the document with all sections, see nature.com/documents/nr-reporting-summary-flat.pdf

Life sciences study design

All studies must disclose on these points even when the disclosure is negative.

Sample size	No sample size calculations were performed. Sample sizes were determined according to established methodologies for working with mammalian oocytes as described in Mogessie, 2020, Meth. Mol. Biol. Each experimental group in an experiment typically consists of a minimum of 8 oocytes per experiment. We have determined through our previously published research studies that these numbers are sufficient to maintain rigor and reproducibility of our data. In most cases, experiments contain sample sizes that exceed this number. Reproducibility and statistical analyses examples can be found in Mogessie and Schuh, 2017; Scheffler et al, 2022, Dunkley and Mogessie. 2023)
Data exclusions	No data were excluded
Replication	All major findings throughout the study were confirmed in a minimum of 3 independent experiments.
Randomization	Randomization was achieved by mixing oocytes isolated from at least two mice. These samples were then divided into different control and experimental groups.
Blinding	Blinding was not employed because the experiments in this study rely on quantitative, objective imaging and biochemical readouts (e.g., fluorescence intensity measurements, chromatid counts, western blot band quantification). In addition, many assays required live manipulation and real-time assessment of oocytes, making blinding impractical. Given the automated, quantitative nature of the analyses and the consistency of the results across multiple independent replicates, we do not believe blinding was necessary to ensure the rigor or reproducibility of our findings

Reporting for specific materials, systems and methods

We require information from authors about some types of materials, experimental systems and methods used in many studies. Here, indicate whether each material, system or method listed is relevant to your study. If you are not sure if a list item applies to your research, read the appropriate section before selecting a response.

Materials & experimental systems

n/a	Involved in the study	
☐	☒	Antibodies
☒	☐	Eukaryotic cell lines
☒	☐	Palaeontology and archaeology
☐	☒	Animals and other organisms
☒	☐	Clinical data
☒	☐	Dual use research of concern
☒	☐	Plants

Methods

n/a	Involved in the study	
☒	☐	ChIP-seq
☒	☐	Flow cytometry
☒	☐	MRI-based neuroimaging

Antibodies

Antibodies used	GFP antibody (1:1000, Novus Bio, NBP2-50059).<br>goat anti-rabbit IgG StarBright Blue 700 secondary antibody (1:5000, Bio-Rad)<br>GFP antibody, 1:100; Roche, 11814460001<br>Alexa Fluor 488–labeled anti-mouse secondary antibody (1:500; Molecular Probes)<br>human anti-centromere antibody, 1:1000 dilution, 15-234, Antibodies Incorporated<br>Alexa Fluor 647–labeled anti-human (1:2000 dilution; Molecular Probes)<br>Rabbit anti-CENP-A, 1: 200 dilution, Cell Signaling, C51A7<br>Alexa Fluor 488–labeled anti-rabbit (1:2000 dilution; Molecular Probes)<br>mouse anti-HEC1, 1:500 dilution, Santa Cruz Biotechnology, sc-135934<br>Alexa Fluor 488–labeled anti-mouse (1:2000 dilution; Molecular Probes)<br>mouse anti-Phospho-Histone H2A.x (Ser139) (1:500, Merck, 05-636)<br>Alexa Fluor 488–labeled anti-mouse (1:2000 dilution; Molecular Probes)
Validation	Anti-GFP Antibody from Roche (Catalog #11814460001) - this antibody was validated by immunofluorescence microscopy for crossreactivity with mClover3 protein but not with mScarlet protein in Fig. S1D, and by assessing localization to chromosome arms as it recognizes endogenous REC8-mClover3 IN Fig. 5A and Fig. S1E.<br>Anti-GFP Antibody from Novusbio (Catalog #NBP2-50059) - antibody was validated in western blotting analyses of PROTAC-mediated dose-dependent degradation of REC8-mClover3 in Fig. S3.<br>Anti-centromere antibody from Antibodies Incorporated (Catalog #15-234) -this antibody was validated by immunofluorescence microscope to assess premature sister chromatid separation and aneuploidyin Fig. 5C, Fig. S2B and Fig. S2D<br>Anti-CENP-A (Cell Signaling) was validated by immunofluorescence microscopy to evaluate CENP-A signal degradation induced by dTAG-13 treatment or CENP-A TRIM-Away in Fig. 6B.<br>Anti-HEC1 antibody from Santa Cruz Biotechnology was validated by immunofluorescence microscopy to assess kinetochore fragmentation following dTAG-13 treatment in Fig. S5A.<br>Anti-Phospho-Histone H2A.x (Ser139) from Merck was validated by immunofluorescence microscopy to assess DNA damage following dTAG-13 treatment in Fig. S5C.

Animals and other research organisms

Policy information about studies involving animals; ARRIVE guidelines recommended for reporting animal research, and Sex and Gender in Research

Laboratory animals	The mouse strains used in this study included REC8-FKBP12F36V-mClover3 and wild-type C57BL/6J (The Jackson Laboratory). Ages were from 6-12 weeks old.
Wild animals	N/A
Reporting on sex	Female mice were used in this study as the research focus is on oocyte meiosis. In one experiment, spermatocytes were collected from from a male mouse for western blotting analyses.
Field-collected samples	N/A
Ethics oversight	All protocols involving mice were approved by the Yale University Institutional Animal Care and Use Committee (approval number 2024-20408). Mice were housed under specific pathogen-free (SPF) conditions with a 12-hour light/dark cycle.

Note that full information on the approval of the study protocol must also be provided in the manuscript.

Plants

Seed stocks	*Report on the source of all seed stocks or other plant material used. If applicable, state the seed stock centre and catalogue number. If plant specimens were collected from the field, describe the collection location, date and sampling procedures.*
Novel plant genotypes	*Describe the methods by which all novel plant genotypes were produced. This includes those generated by transgenic approaches, gene editing, chemical/radiation-based mutagenesis and hybridization. For transgenic lines, describe the transformation method, the number of independent lines analyzed and the generation upon which experiments were performed. For gene-edited lines, describe the editor used, the endogenous sequence targeted for editing, the targeting guide RNA sequence (if applicable) and how the editor was applied.*
Authentication	*Describe any authentication procedures for each seed stock used or novel genotype generated. Describe any experiments used to assess the effect of a mutation and, where applicable, how potential secondary effects (e.g. second site T-DNA insertions, mosiacism, off-target gene editing) were examined.*
