# Supplementary figures and images for: A versatile cohesion manipulation system probes female reproductive age-related egg aneuploidy
Source: Nat Aging. 2025 Nov 3;5(11):2215–27. doi: 10.1038/s43587-025-00997-w (PMC12618256; doi:10.1038/s43587-025-00997-w)

Extended Data Fig.1C

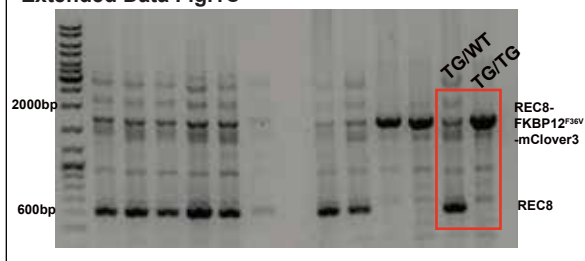

Supplement: Supplementary file 25 — Unprocessed gel. [file 43587_2025_997_MOESM25_ESM.pdf]

Extended Data Fig.3

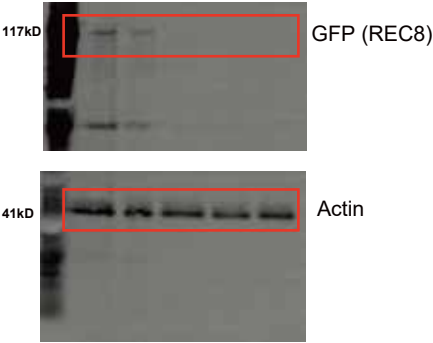

Supplement: Supplementary file 27 — Unprocessed western blots. [file 43587_2025_997_MOESM27_ESM.pdf]
